# A Spiking Neural Network Framework for Robust Sound Classification

**DOI:** 10.3389/fnins.2018.00836

**Published:** 2018-11-19

**Authors:** Jibin Wu, Yansong Chua, Malu Zhang, Haizhou Li, Kay Chen Tan

**Affiliations:** ^1^Department of Electrical and Computer Engineering, National University of Singapore, Singapore, Singapore; ^2^Institute for Infocomm Research, A*STAR, Singapore, Singapore; ^3^Department of Computer Science, City University of Hong Kong, Kowloon Tong, Hong Kong

**Keywords:** spiking neural network, self-organizing map, automatic sound classification, maximum-margin Tempotron classifier, noise robust multi-condition training

## Abstract

Environmental sounds form part of our daily life. With the advancement of deep learning models and the abundance of training data, the performance of automatic sound classification (ASC) systems has improved significantly in recent years. However, the high computational cost, hence high power consumption, remains a major hurdle for large-scale implementation of ASC systems on mobile and wearable devices. Motivated by the observations that humans are highly effective and consume little power whilst analyzing complex audio scenes, we propose a biologically plausible ASC framework, namely SOM-SNN. This framework uses the unsupervised self-organizing map (SOM) for representing frequency contents embedded within the acoustic signals, followed by an event-based spiking neural network (SNN) for spatiotemporal spiking pattern classification. We report experimental results on the RWCP environmental sound and TIDIGITS spoken digits datasets, which demonstrate competitive classification accuracies over other deep learning and SNN-based models. The SOM-SNN framework is also shown to be highly robust to corrupting noise after multi-condition training, whereby the model is trained with noise-corrupted sound samples. Moreover, we discover the early decision making capability of the proposed framework: an accurate classification can be made with an only partial presentation of the input.

## 1. Introduction

Automatic sound classification generally refers to the automatic identification of ambient sounds in the environment. Environmental sounds, complementary to visual cues, informs us of our surrounding environment and is an essential part of our daily life. ASC technologies enable a wide range of applications including, but not limited to content-based sound classification and retrieval (Guo and Li, 2003), audio surveillance (Rabaoui et al., 2008), sound event classification (Dennis et al., 2011) and disease diagnosis (Kwak and Kwon, 2012).

**Figure 1:**
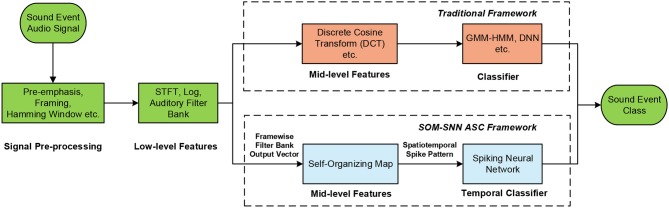
Overview of the proposed SOM-SNN ASC framework, which uses the SOM as a mid-level feature representation of frequency contents in the sound frames, and classifies the spatiotemporal spike patterns using SNNs.

The conventional ASC systems are inspired by automatic speech recognition systems, which typically comprise of acoustic signal pre-processing, feature extraction and classification (Sharan and Moir, 2016). As shown in Figure 1, signal pre-processing can be further sub-categorized into pre-emphasis (high-frequency components are amplified), segmenting (continuous acoustic signals are segmented into overlapping short frames), and windowing (a window function is applied to reduce the effect of spectral leakage). Several feature representations for acoustic signals have been proposed over the years for capturing frequency contents and temporal structures of acoustic signals (Mitrović et al., 2010). The most frequently used features are the Mel-Frequency Cepstral Coefficients (MFCC) (Chu et al., 2009) and Gammatone Cepstral Coefficients (GTCC) (Leng et al., 2012). Both these features mimic the human auditory system, as they are more sensitive to changes in the low-frequency components. These frame-based features are then used to train a GMM-HMM or deep learning models in a classification task.

Despite the significant performance improvement in recent years driven by deep learning models and the abundance of training data, two major challenges remain to prevent the large-scale adoption of such frame-based ASC systems on mobile and wearable devices. First of all, high-performance computing, which typically entails high power consumption, is commonly unavailable on such devices. Secondly, the performance of state-of-the-art GMM-HMM and deep learning models, with MFCC or GTCC feature as input, degrades significantly with increased background noise.

We note that in comparison to existing machine learning techniques, human performs much more efficiently and robustly in various auditory perception tasks, whereby different frequency components of the acoustic signal are asynchronously encoded using sparse and highly parallel spiking impulses. Remarkably, even though spiking impulses in biological neural systems are transmitted at rates of several orders of magnitude slower than signals in modern transistors, humans perceive complex audio scenes with much lower energy consumption (Merolla et al., 2014). Moreover, human learn to distinguish sounds with only sparse supervision, currently formulated as zero-shot or one-shot learning (Fei-Fei et al., 2006; Palatucci et al., 2009) in machine learning. These observations of human auditory perception motivate us to explore and design a biologically plausible event-based ASC system.

Event-based computation, as observed in the human brain and nervous systems, relies on asynchronous and highly parallel spiking events to efficiently encode and transmit information. In contrast to traditional frame-based machine vision and auditory systems, event-based biological systems represent and process information in a much more energy efficient manner whereby energy is only consumed during spike generation and transmission. Spiking neural network (SNN) is one such class of neural networks motivated by event-based computation. For training the SNN on a temporal pattern classification task, many temporal learning rules have been proposed. Depending on how the error function is formulated, they can be categorized into either spike-time based (Ponulak and Kasiński, 2010; Yu et al., 2013a) or membrane-potential based (Gütig and Sompolinsky, 2006; Gütig, 2016; Zhang et al., 2017). For spike-time based learning rules, the main objective is to minimize the time difference between the actual and desired output spike patterns by updating the synaptic weights. In contrast, membrane potential based learning rules use the voltage difference between the actual membrane potential and the firing threshold to guide synaptic weight updates.

Recently, there are growing interests in integrating event-based sensors, such as the DVS (Delbrück et al., 2010), DAVIS (Brandli et al., 2014) and DAS (Liu et al., 2014), with event-based neuromorphic processors such as TrueNorth (Merolla et al., 2014) and SpiNNaker (Furber et al., 2013) for more energy efficient applications (Serrano-Gotarredona et al., 2015; Amir et al., 2017).

In this work, we propose a novel SNN framework for automatic sound classification. We adopt a biologically plausible auditory front-end (using logarithmic mel-scaled filter banks that resemble the functionality of the human cochlea) to first extract low-level spectral features. After which, the unsupervised self-organizing map (SOM) (Kohonen, 1998) is used to generate an effective and sparse mid-level feature representation. The best-matching units (BMUs) of the SOM are activated over time and the corresponding spatiotemporal spike patterns are generated, which represent the characteristics of each sound event. Finally, a newly developed Maximum-Margin Tempotron temporal learning rule (membrane-potential based) is used to classify the spike patterns into different sound categories.

This paper furthers our recent research, which focused on speech recognition (Wu et al., 2018a). In this work, we look into the SOM-SNN properties, system architecture and its robustness against noise in a sound event classification task. We also perform a comparative study with the state-of-the-art deep learning techniques. The main contributions of this work are threefold:

• We propose a biologically plausible event-based ASC framework, namely the SOM-SNN. In this framework, the unsupervised SOM is utilized to represent the frequency contents of environmental sounds, while the SNN learns to distinguish these sounds. This framework achieves competitive classification accuracies compared with deep learning and other SNN-based models on the RWCP and TIDIGITS datasets. Additionally, the proposed framework is shown to be highly robust to corrupting noise after multi-condition training (McLoughlin et al., 2015), whereby the model is trained with noise-corrupted sound samples.

• We propose a new Maximum-Margin Tempotron temporal learning rule, which incorporates the Tempotron (Gütig and Sompolinsky, 2006) with the maximum-margin classifier (Cortes and Vapnik, 1995). This newly introduced hard margin ensures a better separation between positive and negative classes, thereby improving the classification accuracy of the SNN classifier.

• We discover the early decision making capability of the proposed SNN-based classifier, which arises naturally from the Maximum-Margin Tempotron learning rule. The earliest possible discriminative spatiotemporal feature is identified automatically in the SNN classifier, and an output spike is immediately triggered by the correct output neuron. Consequently, an input pattern could be classified with high accuracy when only part of it is presented. Under the same test conditions, the SNN-based classifier consistently outperforms other traditional artificial neural networks (ANNs), [i.e., the Recurrent Neural Network (RNN) (Graves et al., 2013) and Long Short-Term Memory (LSTM) (Hochreiter and Schmidhuber, 1997)] in a temporal pattern classification task. It, therefore, shows great potential for real-world applications, whereby acoustic signals maybe intermittently distorted by noise: the classification decision can be robustly made based on the input portion with less distortion.

## 2. Methods

In this section, we first describe the components of the proposed SOM-SNN framework. Next, we present the experiments designed to evaluate the classification performance and noise robustness of the proposed framework. Finally, we compare it with other state-of-the-art ANN- and SNN-based models.

### 2.1. Auditory front-end

Human auditory front-end consists of the outer, middle and inner ear. In the outer ear, sound waves travel through air and arrive at the pinna, which also embeds the location information of the sound source. From the pinna, the sound signals are then transmitted via the ear canal, which functions as a resonator, to the middle ear. In the middle ear, vibrations (induced by the sound signals) are converted into mechanical movements of the ossicles (i.e., malleus, incus, and stapes) through the tympanic membrane. The tensor tympani and stapedius muscles, which are connected to the ossicles, act as an automatic gain controller to moderate mechanical movements under the high-intensity scenario. At the end of the middle ear, the ossicles join with the cochlea via the oval window, where mechanical movements of the ossicles are transformed into fluid pressure oscillations which move along the basilar membrane in the cochlea (Bear et al., 2016).

The cochlea is a wonderful anatomical work of art. It functions as a spectrum analyzer which displaces the basilar membrane at specific locations that correspond to different frequency components in the sound wave. Finally, displacements of the basilar membrane activate inner hair cells via nearby mechanically gated ion channels, converting mechanical displacements into electrical impulse trains. The spike trains generated at the hair cells are transmitted to the cochlear nuclei through dedicated auditory nerves. Functionally, the cochlear nuclei act as filter banks, which also normalize activities of saturated auditory nerve fibers over different frequency bands. Most of the auditory nerves terminate at the cochlear nuclei where sound information is still identifiable. Beyond the cochlear nuclei, in the auditory cortex, it remains unclear how information is being represented and processed (Møller, 2012).

**Figure 2:**
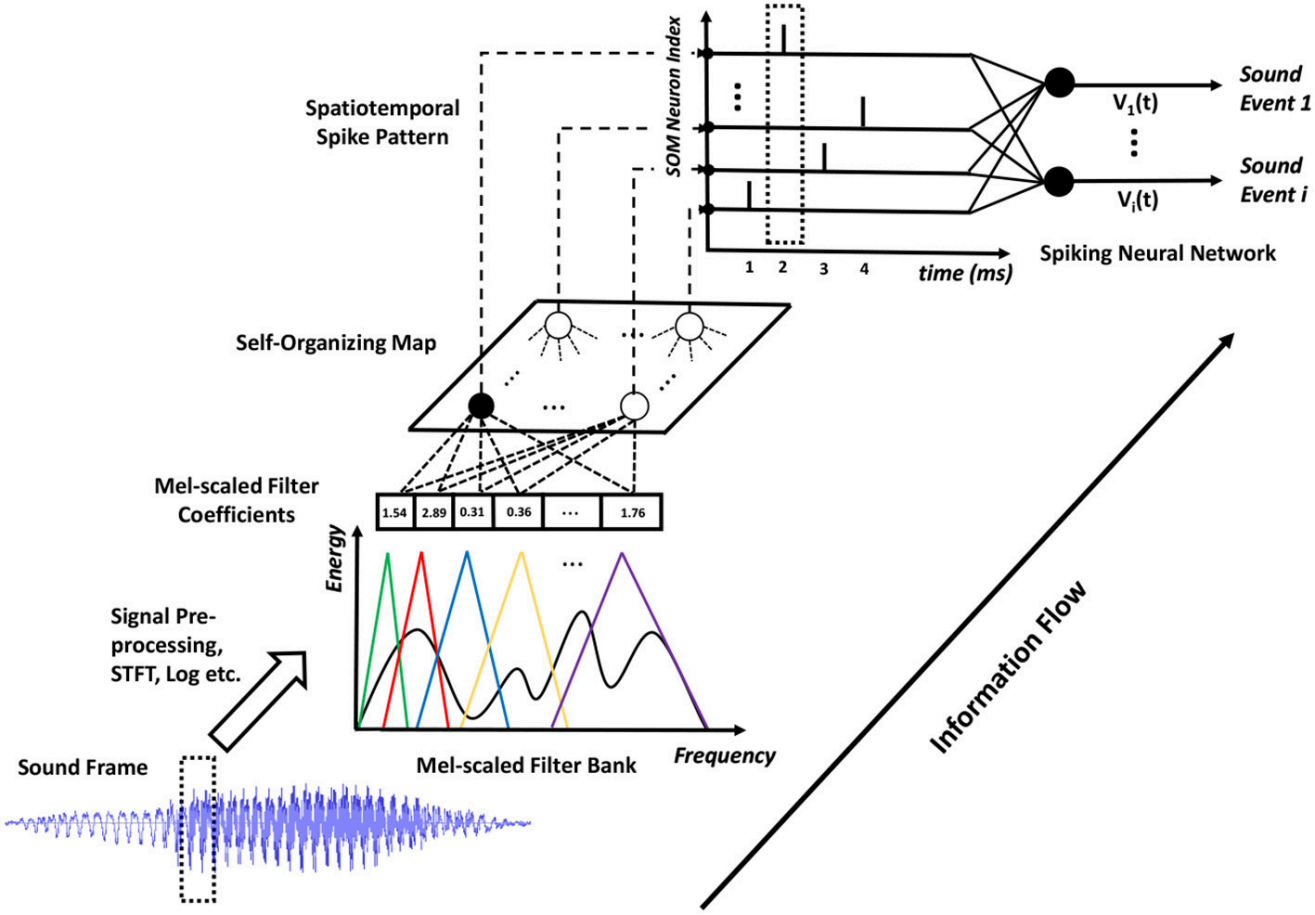
The details of the proposed SOM-SNN ASC framework. The sound frames are pre-processed and analyzed using mel-scaled filter banks. Then, the SOM generates discrete BMU activation sequences which are further converted into spike trains. All such spike trains form a spatiotemporal spike pattern to be classified by the SNN.

The understanding of the human auditory front-end has a significant impact on machine hearing research and inspires many biologically plausible feature representations of acoustic signals, such as the MFCC and GTCC. In this paper, we adopt the MFCC representation. As shown in Figure 2, we pre-processed the sound signals by first applying pre-emphasis to amplify high-frequency contents, then segmenting the continuous sound signals into overlapped frames of suitable length so as to better capture the temporal variations of the sound signal, and finally applying the Hamming window on these frames to reduce the effect of spectral leakage. To extract the spectral contents in the acoustic stimuli, we perform Short-Time Fourier-Transform (STFT) on the sound frames and compute the power spectrum. After that, we apply 20 logarithmic mel-scaled filters on the resulting power spectrum, generating a compressed feature representation for each sound frame. The mel-scaled filter banks emulate the human perception of sound that is more discriminative toward the low frequency as compared to the high frequency components.

### 2.2. Feature representation using SOM

Feature representation is critical in all ASC systems; state-of-the-art ASC systems input low-level MFCC or GTCC features into the GMM-HMM or deep learning models so as to extract higher-level representations. In our initial experiments, we observe that existing SNN temporal learning rules cannot discriminate latency (Yu et al., 2013b) or population (Bohte et al., 2002) encoded mel-scaled filter bank outputs effectively. Therefore, we propose to use the biologically inspired SOM to form a mid-level feature representation of the sound frames. The neurons in the SOM form distinctive synaptic filters that organize themselves tonotopically and compete to represent the filter bank output vectors. Such tonotopically organized feature maps have been found in the human auditory cortex in many physiological experiments (Pantev et al., 1995).

As shown in Figure 2, all neurons in the SOM are fully connected to the filter bank and receive mel-scaled filter outputs (real-valued vectors). The SOM learns acoustic features in an unsupervised manner, whereby two mechanisms: competition and cooperation, guide the formation of a tonotopically organized neural map. During training, the neurons in the SOM compete with each other to best represent the input frame. The best-matching unit (BMU), with its synaptic weight vector closest to the input vector in the feature space, will update its weight vector to become closer to the input vector. Additionally, the neurons surrounding the BMU will cooperate with it by updating their weight vectors to move closer to the input vector. The magnitude of the weight update of neighboring neurons is inversely proportional to its distance to the BMU, effectively facilitating the formation of neural clusters. Eventually, the synaptic weight vectors of neurons in the SOM follow the distribution of input feature vectors and organize tonotopically, such that adjacent neurons in the SOM will have similar weight vectors.

During the evaluation, as shown in Figure 2, the SOM (through the BMU neuron) emits a single spike at each sound frame sampling interval. The sparsely activated BMUs encourage pattern separation and enhance power efficiency. The spikes triggered over the duration of a sound event form a spatiotemporal spike pattern, which is then classified by the SNN into one of the sound classes. The mechanisms of SOM training and testing are provided in Algorithm 1 (see more details Kohonen, 1998). This classical work (Kohonen, 1998) trained the SOM for a phoneme recognition task, which then used a set of hand-crafted rules to link sound clusters of the SOM to actual phoneme classes. In this work, we use an SNN-based classifier to automatically categorize the spatiotemporal spike patterns into different sound events.

**Algorithm 1:**
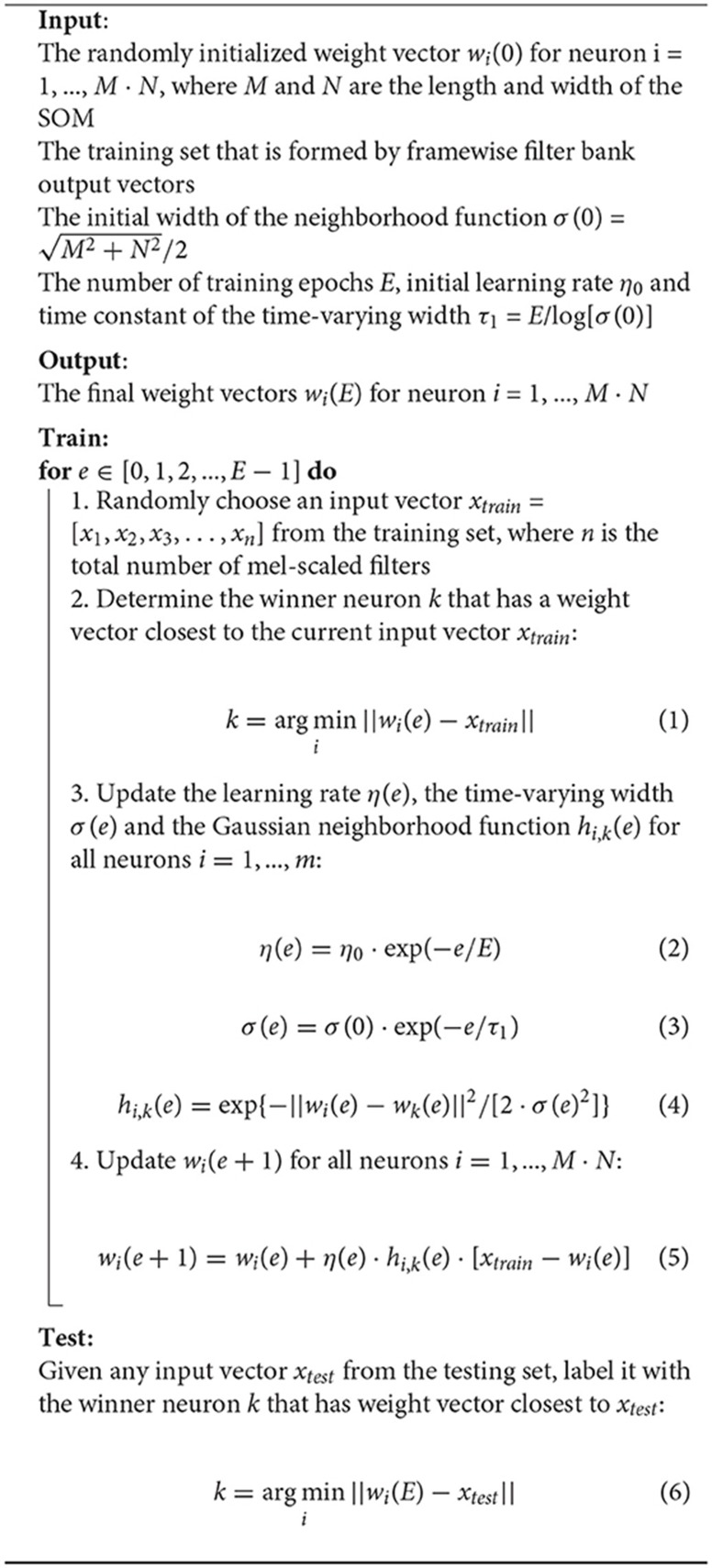


### 2.3. Supervised temporal classification

#### 2.3.1. Neuron model

For the SNN-based temporal classifier, we adopt the leaky integrate-and-fire neuron model (Gütig and Sompolinsky, 2006), which utilizes the kernel function to describe the effect of pre-synaptic spikes on the membrane potential of post-synaptic neurons. When there is no incoming spike, the post-synaptic neuron *i* remains at its resting potential *V*_*rest*_. Each incoming spike from the pre-synaptic neuron *j* at *t*_*j*_ will induce a post-synaptic potential (PSP) on the post-synaptic neuron as described by the following kernel function:

(7) $$\begin{array}{r} K\left(t-t_{j}\right)=K_{0}\left(\exp\left(-\frac{t-t_{j}}{\tau_{m}}\right)-\exp\left(-\frac{t-t_{j}}{\tau_{s}}\right)\right)\theta \left(t-t_{j}\right) \end{array}$$

where *K*_0_ is a normalization factor that ensures the maximum value of the kernel *K*(*t*−*t*_*j*_) is 1. τ_*m*_ and τ_*s*_ correspond to the membrane and synaptic time constants, which jointly determine the shape of the kernel function. In addition, θ(*t*−*t*_*j*_) represents the Heaviside function to ensure that only pre-synaptic spikes emitted before time *t* are considered.

(8) $$\theta \left(x\right)=\left\{ \begin{array}{l} 1,\;if\;x\geq 0\\ 0,\;otherwise \end{array}\right.$$

At time *t*, the membrane potential of the post-synaptic neuron *i* is determined by the weighted sum of all PSPs triggered by incoming spikes before time *t*:

(9) $$\begin{array}{r} V_{i}\left(t\right)=\underset{j}{\sum }w_{ji}\underset{t_{j}<t}{\sum }K\left(t-t_{j}\right)+V_{rest}\;\forall t\in \left[0,T\right] \end{array}$$

where *w*_*ji*_ is the synaptic weight between the pre-synaptic neuron *j* and post-synaptic neuron *i*, and *T* is the duration of the simulation. Whenever the membrane potential *V*_*i*_(*t*) of the post-synaptic neuron *i* reaches the firing threshold, it emits a spike. For the single-spike based classifier used in this work, the membrane potential of the post-synaptic neuron then smoothly relaxes back to *V*_*rest*_ after spiking by shunting all subsequent input spikes (i.e., input spikes arriving after the post-synaptic spike, have no effect on the membrane potential of the post-synaptic neuron). Since these input spikes would not contribute to any learning in the single-spike based classifier, the unnecessary post-spike computations can be safely ignored.

#### 2.3.2. Maximum-margin tempotron learning rule

For the classification of spatiotemporal patterns as illustrated by the SNN in Figure 2, we use a modified version of the biologically plausible Tempotron (Gütig and Sompolinsky, 2006) learning rule to train the classifier, which has been successfully used in several ASC tasks (Dennis et al., 2013; Xiao et al., 2017). The original Tempotron rule is designed for a binary classification task, such that a neuron emits a spike when it observes a spike pattern from its desired class, and remains quiescent otherwise. For a multi-class classification task, we adopt the one-against-all strategy to train one output neuron to respond to each class.

During training, for neuron *i* that represents the *i*th class, we treat all training samples with class label *i* as positive samples, and all others as negative. During testing, we monitor the membrane potential of all output neurons and classify the test sample as follows: (1) If no output neuron fires over the sample duration, we select the output neuron with the highest membrane potential as the correct class. (2) If only a single output neuron fires, the class label corresponding to this neuron is selected. (3) Otherwise, if two or more neurons fire, we label the test sample with the earliest firing neuron, which signals the detection of the earliest local discriminative feature (a property of the Tempotron).

The Tempotron learning rule follows a stochastic gradient descent method for synaptic weight updates: the desired output neuron triggers a weight update whenever it fails to fire on samples with matching class label or when the wrong output neurons fire erroneously on samples from other classes. When the desired output neuron *i* fails to fire, long-term potentiation (LTP) update with cost function *V*_*thr*_ - $V_{t_{i}^{\max}}$ is triggered. Similarly, long-term depression (LTD) update with cost function $V_{t_{i}^{\max}}$ - *V*_*thr*_ is triggered when the wrong output neuron fires erroneously. The Tempotron update rule is defined as follows:

(10) $$\Delta w_{ij}=\left\{ \begin{array}{l} \lambda \sum_{t_{j}^{\left(f\right)}<t_{i}^{\text{max}}}K\left(t_{i}^{\text{max}}-t_{j}^{\left(f\right)}\right),\;if\;LTP\\ -\lambda \sum_{t_{j}^{\left(f\right)}<t_{i}^{\text{max}}}K\left(t_{i}^{\text{max}}-t_{j}^{\left(f\right)}\right),\;if\;LTD\\ 0,\;otherwise \end{array}\right.$$

where λ denotes a constant learning rate and $t_{i}^{\max}$ refers to the time instant when the postsynaptic neuron *i* reaches its maximum membrane potential over the pattern duration. The $t_{j}^{\left(f\right)}$ are spike times of spike emitted by the pre-synaptic neuron *j*. The synaptic weights are only updated at the time instant of $t_{i}^{\max}$. For LTD weight update, the $t_{i}^{\max}$ is also the spike time since the post-spike computations are ignored.

Inspired by the maximum-margin classifier (Cortes and Vapnik, 1995), we introduce a hard margin Δ to the *V*_*thr*_ and denote the new learning rule as the Maximum-Margin Tempotron. During the training phase, the Δ term is either added to or deducted from the *V*_*thr*_ of the desired or wrong output neurons, respectively. Consequently, for the desired neuron *i*, a spike is generated at *t* if

(11) $$\begin{array}{r} V_{i}\left(t\right)=V_{thr}+\Delta \;and\;\frac{d}{dt}V_{i}\left(t\right)>0 \end{array}$$

For the other (wrong) neurons, a spike is generated if

(12) $$\begin{array}{r} V_{i}\left(t\right)=V_{thr}-\Delta \;and\;\frac{d}{dt}V_{i}\left(t\right)>0 \end{array}$$

The desired output neuron will fire only when it has observed strong evidence that causes its *V*_*t*_*max*__ to rise above *V*_*thr*_ by a margin of Δ. Similarly, the other neurons will be discouraged to fire and maintain its membrane potential by a margin Δ below *V*_*thr*_. This additional margin Δ imposes a harder constraint during training and encourages the SNN classifier to find more discriminative features in the input spike patterns. Therefore, during testing, when the hard margin Δ is removed from *V*_*i*_(*t*) as described in Equation (9), the neurons are encouraged to respond with the desired spiking activities. This strategy helps to prevent overfitting and improves classification accuracy.

### 2.4. Multi-condition training

Although state-of-the-art deep learning based ASC models perform reasonably well under the noise-free condition, it remains a challenging task for these models to recognize sound robustly in noisy real-world environments. To address this challenge, we investigated training the proposed SOM-SNN model with both clean and noisy sound data, as per the multi-condition training strategy.

The motivation for such an approach is that with training samples collected from different noisy backgrounds, the trained model will be encouraged to identify the most discriminative features and become more robust to noise. This methodology has been proven to be effective for Deep Neural Network (DNN) and SVM models under the high noise condition, with some trade-off in performance for clean sound data (McLoughlin et al., 2015). Here, we investigate its generalizability to SNN-based temporal classifiers under noisy environments.

### 2.5. Training and evaluation

Here, we first introduce two standard benchmark datasets used to evaluate the classification accuracies of the proposed SOM-SNN framework, which are made up of environmental sounds and human speech. After which, we describe the experiments conducted on the RWCP dataset to evaluate model performance pertaining to the effectiveness of feature representation using the SOM, early decision making capability and noise robustness of the classifier.

#### 2.5.1. Evaluation datasets

The Real World Computing Partnership (RWCP) (Nishiura and Nakamura, 2002) sound scene dataset was recorded in a real acoustic environment at a sampling rate of 16 kHz. For a fair comparison with other SNN-based systems (Dennis et al., 2013; Xiao et al., 2017), we used the same 10 sound event classes from the dataset: “cymbals,” “horn,” “phone4,” “bells5,” “kara,” “bottle1,” “buzzer,” “metal15,” “whistle1,” “ring.” The sound clips were recorded as isolated samples with duration of 0.5s to 3s at high SNR. There are also short lead-in and lead-out silent intervals in the sound clips. We randomly selected 40 sound clips from each class, of which 20 are used for training and the remaining 20 for testing, giving a total of 200 training and 200 testing samples.

The TIDIGITS (Leonard and Doddington, 1993) dataset consists of reading digit strings of varying lengths, and the speech signals are sampled at 20 kHz. The TIDIGITS dataset is a publicly available dataset from the Linguistic Data Consortium, which is one of the most commonly available speech datasets used for benchmarking speech recognition algorithms. This dataset consists of spoken digit utterances from 111 male and 114 female speakers. We used all of the 12,373 continuous spoken digit utterances for the SOM training and the rest of the 4,950 isolated spoken digit utterances for the SNN training and testing. Each speaker contributes two isolated spoken digit utterances for all 11 classes (i.e., “zeros” to “nine” and “oh”). We split the isolated spoken digit utterances randomly with 3,950 utterances for training and the remaining 1,000 utterances for testing.

#### 2.5.2. SOM-SNN framework

The SOM-SNN framework, as shown in Figure 2, consists of three processing stages organized in a pipeline. These stages are trained separately and then evaluated in a single, continuous process. For the auditory front-end, we segment the continuous sound samples into frames of 100 ms length with 50 ms overlap between neighboring frames for the RWCP dataset. In contrast, we use a frame length of 25 ms with 10 ms overlap for the TIDIGITS dataset. These values are determined empirically to sufficiently discriminate the signals without excessive computational load. We utilize 20 mel-scaled filters for the spectral analysis, ranging from 200 to 8,000 Hz and 200 to 10,000 Hz respectively for the RWCP and TIDIGITS datasets. The number of filters is again empirically determined, such that more filters do not improve classification accuracy.

For feature representation learning in the SOM, we utilize the SOM available in the MATLAB Neural Network Toolbox. The Euclidean distance is used to determine the BMUs, which are subsequently converted into spatiotemporal spike patterns. The output spikes from the SOM are generated per sound frame, with an interval as determined by the frame shift (i.e., 50 ms for RWCP dataset and 15 ms for TIDIGITS dataset). We study the effect of different hyperparameters including SOM map size, number of training epochs and number of activated neurons per incoming frame. Their effects on classification accuracy are presented in section 3.3.

We initialize the SNN by setting the threshold *V*_*thr*_, the hard margin Δ and learning rate λ to 1.0, 0.5 and 0.005 respectively. The time constants of the SNN have determined empirically such that the PSP duration is optimal for the particular dataset, and we set τ_*m*_ to 750, 225 ms and τ_*s*_ to 187.5, 56.25 ms for the RWCP and TIDIGITS datasets, respectively. We train all the SNNs for 10 epochs by when convergence is observed. The initial weights for the neurons in the SNN classifier are drawn randomly from the Gaussian distribution with a mean of 0 and standard deviation of 10^−3^. Parameters used in all our experiments are as above unless otherwise stated.

#### 2.5.3. Traditional artificial neural networks

To facilitate comparison with other traditional ANN models trained on the RWCP dataset, we implement four common neural network architectures, namely the Multi-Layer Perceptron (MLP) (Morgan and Bourlard, 1990), the Convolutional Neural Network (CNN) (Krizhevsky et al., 2012), the Recurrent Neural Network (RNN) (Graves et al., 2013) and the Long Short-Term Memory (LSTM) (Hochreiter and Schmidhuber, 1997) using the Pytorch library. For a fair comparison, we implement the MLP with 1 hidden layer of 500 ReLU units, and the CNN with two convolution layers of 128 feature maps each followed by 2 fully-connected layers of 500 and 10 ReLU units. The input frames to the MLP and CNN are concatenated over time into a spectrogram image. Since the number of frames for each sound clip varies from 20 to 100 and cannot be processed directly by the MLP or CNN, we bilinearly rescale these spectrogram images into a consistent dimension of 20 × 64.

We implement both the RNN and LSTM with two hidden layers containing 100 hidden units each, and a dropout layer with a probability of 0.5 is applied after the first hidden layer to prevent overfitting. The input to the RNN and LSTM are the 20-dimensional filter bank output vectors. The weights for all networks are initialized with orthogonal conditions as suggested in (Saxe et al., 2013). The deep learning networks are trained with the cross-entropy criterion and optimized using the Adam (Kingma and Ba, 2014) optimizer. The learning rate is decayed to 99% of the original value after every epoch, and all networks are trained for 100 epochs, except for the CNN (50 epochs), by when convergence is observed. Simulations are repeated 10 times for each model, with random weight initialization.

To study the synergy between SOM and deep learning models (i.e., RNN and LSTM), we use the mid-level features of the SOM as inputs to train the RNN and LSTM, respectively denoted as SOM-RNN and SOM-LSTM. These features are obtained by converting the BMU that corresponds to each sound frame into a one-hot vector and concatenating them over time to form a sparse representation of each sound clip. We trained the SOM-RNN and SOM-LSTM models with the same set-up as the RNN and LSTM mentioned above.

#### 2.5.4. Noise robustness evaluation

##### 2.5.4.1. Environmental noise

We generate noise-corrupted sound samples by adding “Speech Babble" background noise from the NOISEX-92 dataset (Varga and Steeneken, 1993) to the clean RWCP sound samples. This selected background noise represents a non-stationary noisy environment with predominantly low-frequency contents, hence making a fair comparison with the noise robustness tests performed in LSF-SNN (Dennis et al., 2013) and LTF-SNN models (Xiao et al., 2017). For each training or testing sound sample, a random noise segment of the same duration is selected from the noise file and added at 4 different SNR levels of 20, 10, 0 and -5 dB separately, giving a total of 1,000 training and 1,000 testing samples. The SNR ratio is calculated based on the energy level of each sound sample and the corresponding noise segment in our experiments. Training is performed over the whole training set, while the testing set is evaluated separately at different SNR levels.

We perform multi-condition training on all the MLP, CNN, RNN, LSTM and SOM-SNN models. Additionally, we also conduct experiments whereby the models are trained with clean sound samples but tested with noise-corrupted samples (the mismatched condition).

##### 2.5.4.2. Neuronal noise

We also consider the effect of neuronal noise which is known to exist in the human brain, emulated by spike jittering and deletion. Given that the human auditory system is highly robust to these noises, it motivates us to investigate the performance of the proposed framework under such noisy conditions.

For spike jittering, we add Gaussian noise with zero mean and standard deviation σ to the spike timing *t* of all input spikes entering the SNN classifier. The amount of jitter is determined by σ which we sweep from 0.1 *T* to 0.8 *T*, where *T* is the spike generation period. In addition, we also consider spike deletion, where a certain fraction of spikes are corrupted by noise and not delivered to the SNN. For both types of neuronal noise, we trained the model without any noise and then tested it with jittered (of varying standard deviation σ) or deleted (of varying ratio) input spike trains.

## 3. Results

In this section, we first present the classification results of the proposed SOM-SNN framework for the two benchmark datasets and then compare them with other baseline models. Next, we discuss its early decision-making capability, the effectiveness of using the SOM for feature representation and its underlying hyperparameters, as well as the key differences between the feedforward SNN-based and RNN-based systems for a temporal classification task. Finally, we demonstrate the improved classification capability of the modified Maximum-Margin Tempotron learning rule and the robustness of the framework against environmental and neuronal noises.

### 3.1. Classification results

#### 3.1.1. RWCP dataset

As shown in Table 1, the SOM-SNN model achieved a test accuracy of 99.60%, which is competitive compared with other deep learning and SNN-based models. As described in the experimental set-up, the MLP and CNN models are trained using spectrogram images of fixed dimensions, instead of explicitly modeling the temporal transition of frames. Despite their high accuracy on this dataset, it may be challenging to use them for classifying sound samples of long duration; the temporal structures will be affected inconsistently due to the necessary rescaling of the spectrogram images (Gütig and Sompolinsky, 2009). On the other hand, the RNN and LSTM models capture the temporal transition explicitly. These models are however hard to train for long sound samples due to the vanishing and exploding gradient problem (Greff et al., 2017).

**Table 1:** Comparison of the classification accuracy of the proposed SOM-SNN framework against other ANNs and SNN-based frameworks on the RWCP dataset.

**Model**	**Accuracy (%)**
MLP	99.45
CNN	99.85
RNN	95.35
LSTM	98.40
SOM-RNN	97.20
SOM-LSTM	98.15
LSF-SNN (Dennis et al., 2013)	98.50
LTF-SNN (Xiao et al., 2017)	97.50
SOM-SNN (ReSuMe)	97.00
SOM-SNN (Maximum-Margin Tempotron)	99.60

*The average results over 10 experimental runs with random weight initialization are reported*.

LSF-SNN (Dennis et al., 2013) and LTF-SNN (Xiao et al., 2017) classify the sound samples by first detecting the spectral features in the power spectrogram, and then encoding these features into a spatiotemporal spike pattern for classification by a SNN classifier. In our framework, the SOM is used to learn the key features embedded in the acoustic signals in an unsupervised manner, which is more biologically plausible. Neurons in the SOM become selective to specific spectral features after training, and these features learned by the SOM are more discriminative as shown by the superior SOM-SNN classification accuracy compared with the LSF-SNN and LTF-SNN models.

#### 3.1.2. Tidigits dataset

As shown in Table 2, it is encouraging to note that the SOM-SNN framework achieves an accuracy of 97.40%, outperforming all other bio-inspired systems on the TIDIGITS dataset. In Anumula et al. (2018), Abdollahi and Liu (2011), and Neil and Liu (2016), novel systems are designed to work with spike streams generated directly from the AER silicon cochlea sensor. This event-driven auditory front-end generates spike streams asynchronously from 64 bandpass filters spanning over the audible range of the human cochlea. Anumula et al. (Abdollahi and Liu, 2011) provide a comprehensive overview of the asynchronous and synchronous features generated from these raw spike streams, once again highlighting the significant role of discriminative feature representation in speech recognition tasks.

**Table 2:** Comparison of the classification accuracy of the proposed SOM-SNN framework against other baseline frameworks on the TIDIGITS dataset.

**Model**	**Accuracy (%)**
Single-layer SNN and SVM (Tavanaei and Maida, 2017a)^*a*^	91.00
Spiking CNN and HMM (Tavanaei and Maida, 2017b)^*a*^	96.00
AER Silicon Cochlea and SVM (Abdollahi and Liu, 2011)^*b*^	95.58
AER Silicon Cochlea and Deep RNN (Neil and Liu, 2016)^*b*^	96.10
AER Silicon Cochlea and Phased LSTM (Anumula et al., 2018)^*b*^	91.25
Liquid State Machine (Zhang et al., 2015)^*c*^	92.30
MFCC and GRU RNN (Anumula et al., 2018)^*c*^	97.90
SOM and SNN (this work)^*c*^	97.40

a *Evaluate on the Aurora dataset which was developed from the TIDIGITS dataset*.

b *The data was collected by playing the audio files from the TIDIGITS dataset to the AER Silicon Cochlea Sensor*.

c *Evaluate on the TIDIGITS dataset*.

Tavanaei et al. (Tavanaei and Maida, 2017a,b) proposes two biologically plausible feature extractors constructed from SNNs trained using the unsupervised spike-timing-dependent plasticity (STDP) learning rule. The neuronal activations in the feature extraction layer are then transformed into a real-valued feature vector and used to train a traditional classifier, such as the HMM or SVM models. In our work, the features are extracted using the SOM and then used to train a biologically plausible SNN classifier. These different biologically inspired systems represent an important step toward an end-to-end SNN-based automatic speech recognition system.

We note that the traditional RNN based system offers a competitive accuracy of 97.90% (Anumula et al., 2018); our proposed framework, however, is fundamentally different from traditional deep learning approaches. It is worth noting that the network capacity and classification accuracy of our framework can be further improved using multi-layer SNNs.

### 3.2. Early decision making capability

**Figure 3:**
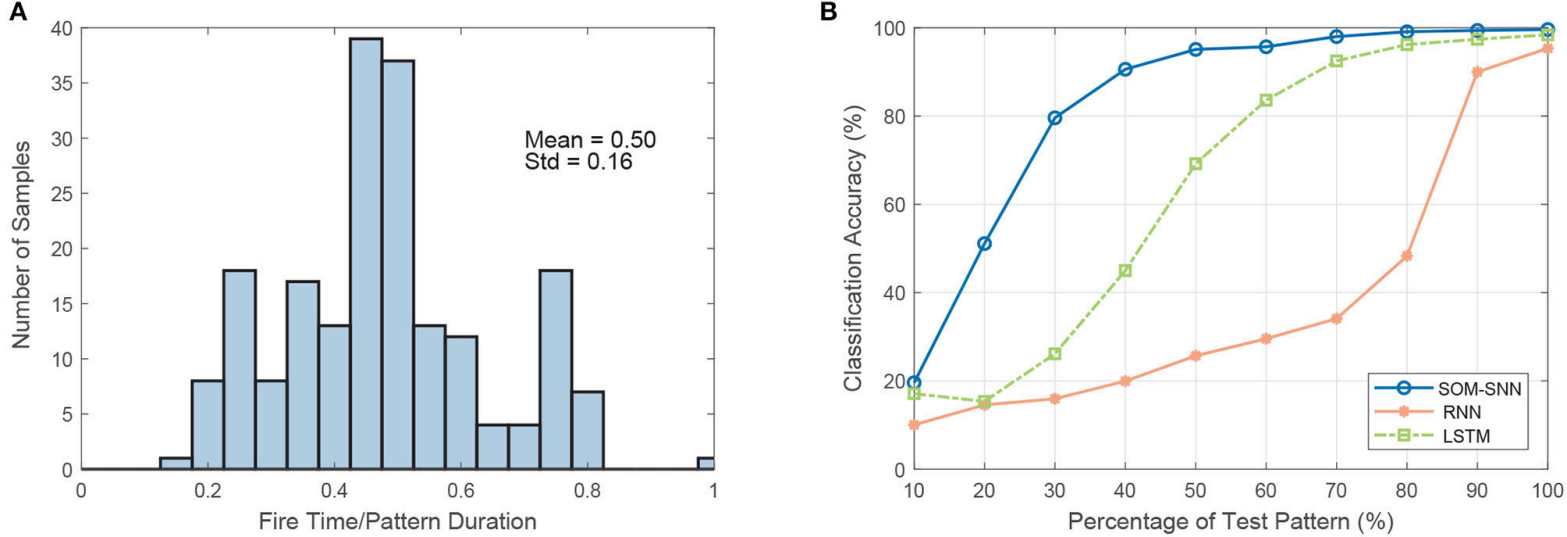
The demonstration of the early decision making capability of the SNN-based classifier. **(A)** The distribution of the number of samples as a function of the ratio of decision time (spike timing) to sample duration on the RWCP test dataset. On average, the SNN-based classifier makes the classification decision when only 50% of the pattern is presented. **(B)** Test accuracy as a function of the percentage of test pattern input to different classifiers (classifiers are trained with full training patterns).

We note that the SNN-based classifier can identify temporal features within the spatiotemporal spike pattern and generate an output spike as soon as enough discriminative evidence is accumulated. This cumulative decision-making process is more biologically plausible, as it mimics how human makes decisions. A key benefit of such a decision-making process is low latency. As shown in Figure 3A, the SNN classifier makes a decision before the whole pattern has been presented. On average, the decision is made when only 50% of the input is presented.

Additionally, we conduct experiments on the SOM-SNN, RNN, and LSTM models, whereby they are trained on the full input patterns but tested with only a partial presentation of the input. The training label is provided to the RNN and LSTM models at the end of each training sequence by default as it is not clear beforehand when enough discriminative features have been accumulated. Likewise, the training labels are provided at the end of input patterns for the SNN classifier. For testing, we increase the duration of the test input pattern presented from 10 to 100% of the actual duration, starting from the beginning of each pattern. As shown in Figure 6B, the classification accuracy as a function of the input pattern percentage increases more rapidly for the SNN model. It achieves a satisfactory accuracy of 95.1% when only 50% of the input pattern is presented, much higher than the 25.7 and 69.2% accuracy achieved by the RNN and LSTM models respectively. For the RNN and LSTM models to achieve early decision-making capability, one may require that the models be trained with partial inputs or output labels provided at every time-step. Therefore, SNN-based classifiers demonstrate great potential for real-time temporal pattern classification, compared with state-of-the-art deep learning models such as the RNN and LSTM.

### 3.3. Feature representation of the SOM

**Figure 4:**
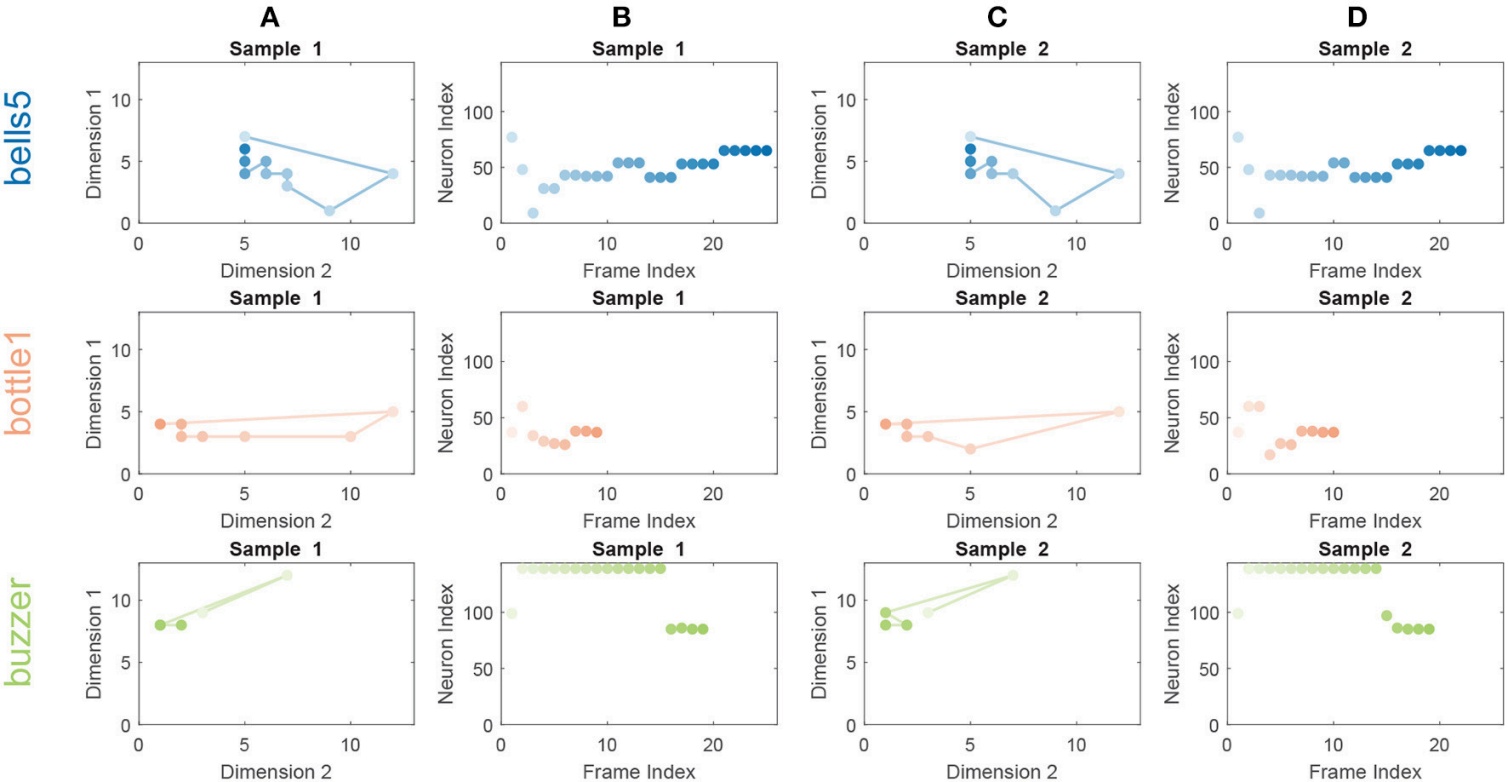
BMU activation trajectories of the SOM **(A,C)** and BMU activation sequences **(B,D)** for randomly selected sound samples from classes “bell5” (Row 1), “bottle1” (Row 2) and “buzzer” (Row 3) of a trained 12 × 12 SOM on the RWCP dataset. For BMU activation trajectories, the lines connect activated BMUs from frame to frame. The activated BMUs are highlighted from light to dark over time. For BMU activation sequences, the neurons of the SOM are enumerated along the y-axis and color matched with neurons in the BMU activation trajectories. The low intra-class variability and high inter-class variability for the BMU activation trajectories and sequences are observed.

**Figure 5:**
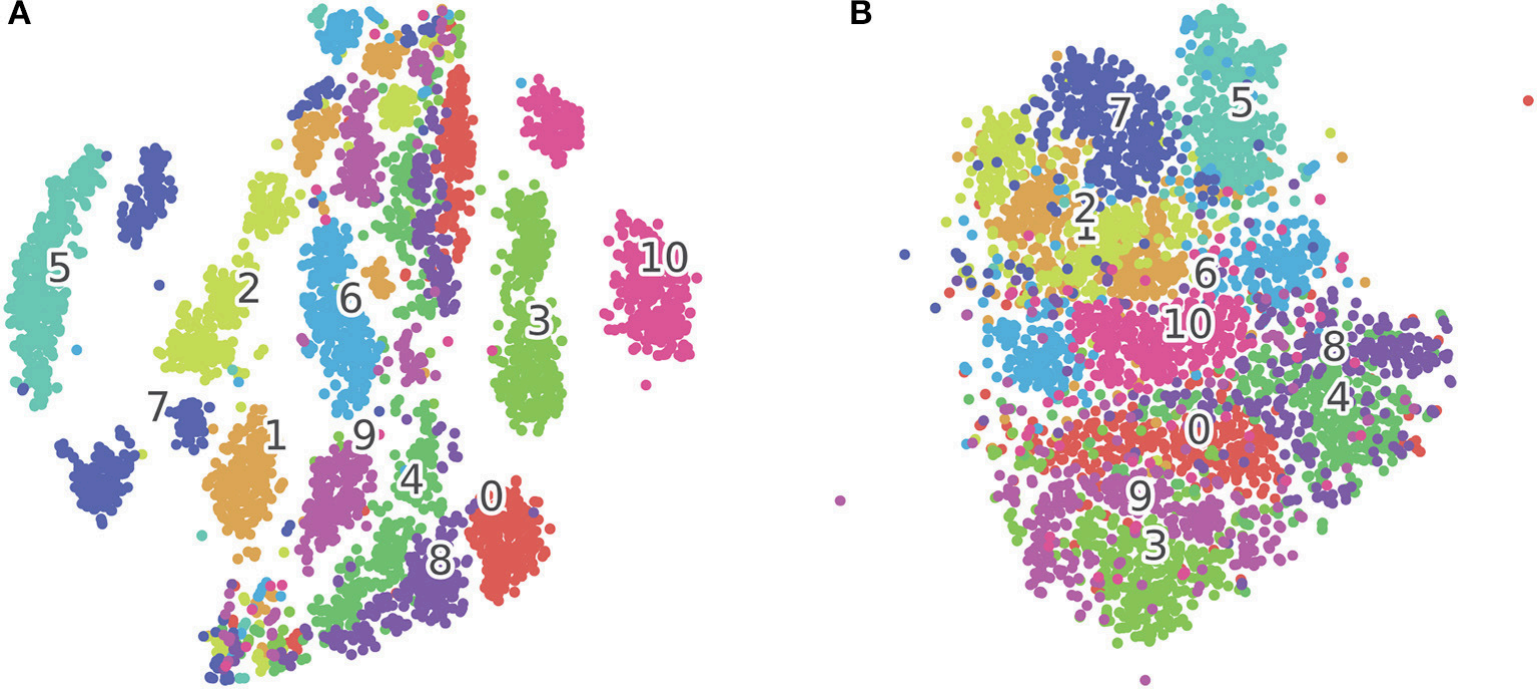
**(A)** tSNE clustering for concatenated input vectors entering the SOM. **(B)** tSNE clustering for BMU trajectories output from the SOM. Each dot on the figure corresponds to one test sample in the TIDIGITS dataset, the numbers in the figure correspond to class centroids. The samples (e.g., Class 7 and 10) within the same class get closer after being processed by the SOM as shown in this 2D visualization.

To visualize the features extracted by the SOM, we plot the BMU activation sequences and their corresponding trajectories on the SOM for a set of randomly selected samples from class “bell5,” “bottle1,” and “buzzer” in Figure 4. We observe low intra-class variability and high inter-class variability in both the BMU activation trajectories and sequences, which are highly desirable for pattern classification. Furthermore, we perform tSNE clustering on the concatenated input vectors entering the SOM and the BMU trajectories generated by the SOM. In Figure 5A (input vectors entering the SOM), it can be seen that samples from the same class are distributed over several clusters in 2D space (e.g., class 7, 10). The corresponding BMU vectors, however, merge into a single cluster as shown in Figure 5B, suggesting lower intra-class variability achieved by the SOM. The class boundaries for the BMU trajectories may now be drawn as shown in Figure 5B, suggesting high inter-class variability. The outliers in Figure 5B maybe an artifact due to the uniform rescaling performed on BMU trajectories, a necessary step for tSNE clustering.

We note that the time-warping problem exists in the BMU activation sequences, whereby the duration of sensory stimuli fluctuates from sample to sample within the same class. However, the SNN-based classifier is robust to such fluctuations as shown in the classification results. The decision to fire for a classifying neuron is made based on a time snippet of the spiking pattern; such is the nature of the single spike-based temporal classifier. As long as the BMU activation sequence stays similar, duration fluctuations of input sample will not affect the general trajectory of the membrane potential in each output neuron; the right classification decision, therefore, can be guaranteed. Hence, those outliers in Figure 5B underlying the time-warping problem may not necessarily lead to poor classification.

To investigate whether the feature dimension reduction of the SOM is necessary for the SNN classifier to learn different sound categories, we performed experiments that directly input the spike trains of the latency-encoded (20 neurons) (Yu et al., 2013b) or population-encoded (144 neurons) (Bohte et al., 2002) mel-scaled filter bank outputs into the SNN for classification. We find that the SNN classifier is unable to classify such low-level spatiotemporal spike patterns, and only achieve 10.2 and 46.5% classification accuracy for latency- and population-encoded spike patterns, respectively. For both latency- and population-encoded spike patterns, as all encoding neurons spike in every sound frame, albeit with different timing, the synaptic weights therefore either all strengthen or all weaken in the event of misclassification as defined in the Tempotron learning rule. Such synchronized weight updates make it challenging for the SNN classifier to find discriminative features embedded within the spike pattern.

As summarized in the section 1, the learning rules for the SNN can be categorized into either membrane-potential based or spike-time based; the Maximum-Margin Tempotron learning rule belongs to the former. To study the synergy between the SOM-based feature representation and spike-time based learning rule, we conducted an experiment using the ReSuMe (Ponulak and Kasiński, 2010) learning rule to train the SNN classifier. For a fair comparison with the Maximum-Margin Tempotron learning rule, we use one output neuron to represent each sound class and each neuron has a single desired output spike. To determine the desired spike timing for each output neuron, we first present all training spiking patterns from the corresponding sound class to the randomly initialized SNN; and monitor the membrane potential trace of the desired output neuron during the simulation. We note the time instant when the membrane potential trace reaches its maximum (denoted as *T*_*max*_) for each sound sample, revealing the most discriminative local temporal feature. We then use the mean of *T*_*max*_ across all 20 training samples as the desired output spike time. As shown in Table 1, the SNN trained with ReSuMe rule achieves a classification accuracy of 97.0%, which is competitive with other models. This, therefore, demonstrates the compatibility of features extracted by the SOM and spike-time based learning rules, whereby the intra-class variability of sound samples is circumvented by SOM feature extraction such that a single desired spike time for each class suffices.

We note that the SOM functions as an unsupervised sparse feature extractor that provides useful, discriminative input to downstream ANN classifiers. As shown in Table 1, the classification accuracy of the SOM-RNN model is better than that of the RNN model alone, and the accuracy of the SOM-LSTM model is also comparable to that of the LSTM model. Additionally, we also notice faster training convergence for both the SOM-RNN and SOM-LSTM models compared to those without the SOM, requiring approximately 25% less number of epochs. This observation may be best explained by the observations made in Figure 4, whereby only a subset of the SOM neurons are involved in the spiking patterns of any sound sample (with low intra-class variability and high inter-class variability) which in itself is highly discriminative.

**Figure 6:**
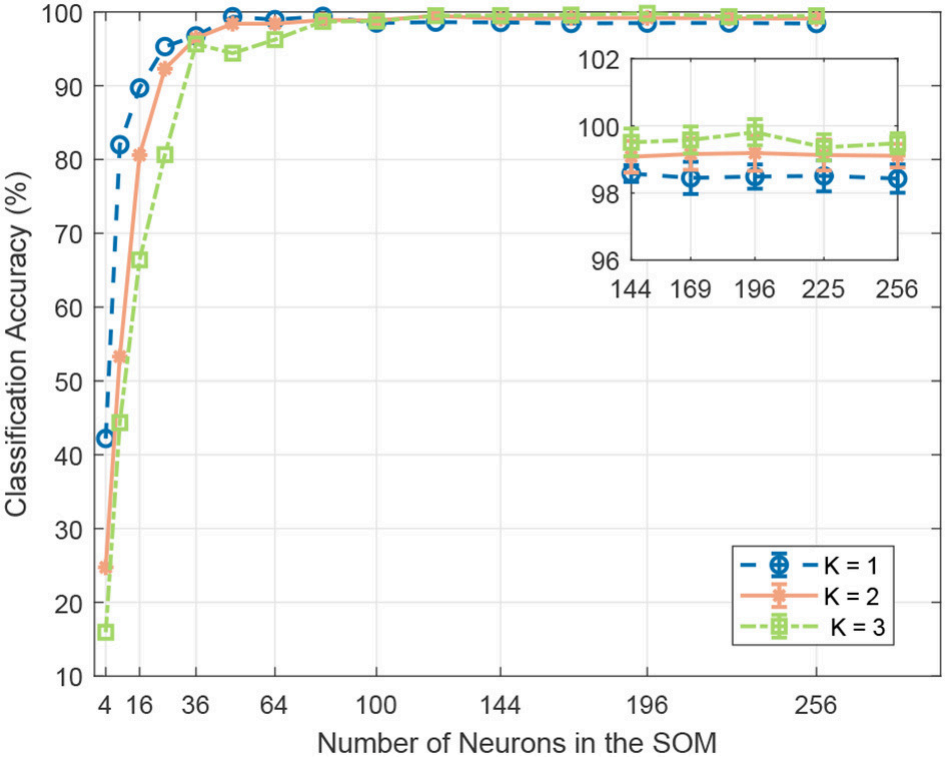
The effect of the SOM neural map size and number of BMUs per frame on classification accuracy. A larger neural map can capture more feature variations and generate more discriminative spiking patterns for different sound events. However, the accuracy plateaus once the number of neurons exceeds 121. As shown in the inset, for neural maps of size above 121, increasing the number *K* of BMUs for each frame enhances system robustness with redundancy and improves classification accuracy.

To analyze the effect of different hyperparameters in the SOM on classification accuracy, we perform the following experiments:

**Neural Map Size**. We sweep the SOM neural map size from 2 × 2 to 16 × 16. As shown in Figure 6, we notice improved SNN classification accuracy with larger neural map, which suggests that a larger SOM captures more discriminative features and therefore generates more discriminative spiking patterns for different sound classes. However, the accuracy plateaus once the number of neurons exceeds 120. We suspect that with more neurons the effect of the time-warping problem starts to dominate, leading to more misclassification. Hence, the optimum neural map size has to be empirically determined.

**Number of Training Epochs**. We sweep the number of training epochs used for the SOM from 100 to 1,000 with an interval of 100. We observe improvements in classification accuracy of the SNN classifier, with more training epochs of the SOM, which plateaus at 400 for the RWCP dataset.

**Number of Activated Neurons**. We perform experiments with different number of activated output neurons *K* = [1, 2, 3] for each sound frame. Specifically, the distances between the SOM output neurons' synaptic weight vectors and the input vector are computed, and the top *K* neurons with the closest weight vectors will emit a spike. The neural map sizes are swept from 2 × 2 to 16 × 16, with number of training epochs fixed at 400. As shown in Figure 6, with more activated output neurons in the SOM, the SNN achieves lower classification accuracy for neural map size below 100, while achieving higher accuracy for neural map size larger than that. It can be explained by the fact that for smaller neural maps, given the same number of feature clusters, fewer neurons are allocated to each cluster. Now, with more activated neurons per frame, either fewer clusters can be represented, or the clusters are now less distinguishable from each other. Either way, inter-class variability is reduced, and classification accuracy is adversely affected. This capacity constraint is alleviated with a larger neural map, whereby neighboring neurons are usually grouped into a single feature cluster. As shown in the inset of Figure 6, for neural map size larger than 100, more activated neurons per frame improves the feature representation with some redundancy and lead to better classification accuracy. However, it should be noted that with more activated neurons per frame, there are more output spikes generated in the SOM, hence increasing energy consumption. Therefore, a trade-off between classification accuracy and energy consumption has to be made for practical applications.

### 3.4. Tempotron learning rule with hard maximum-margin

As described in section 2, we modify the original Tempotron learning rule by adding a hard margin Δ to the firing threshold *V*_*thr*_. With this modification, we note that the classification accuracy of the SNN increases by 2% consistently with the same SOM dimensions.

**Figure 7:**
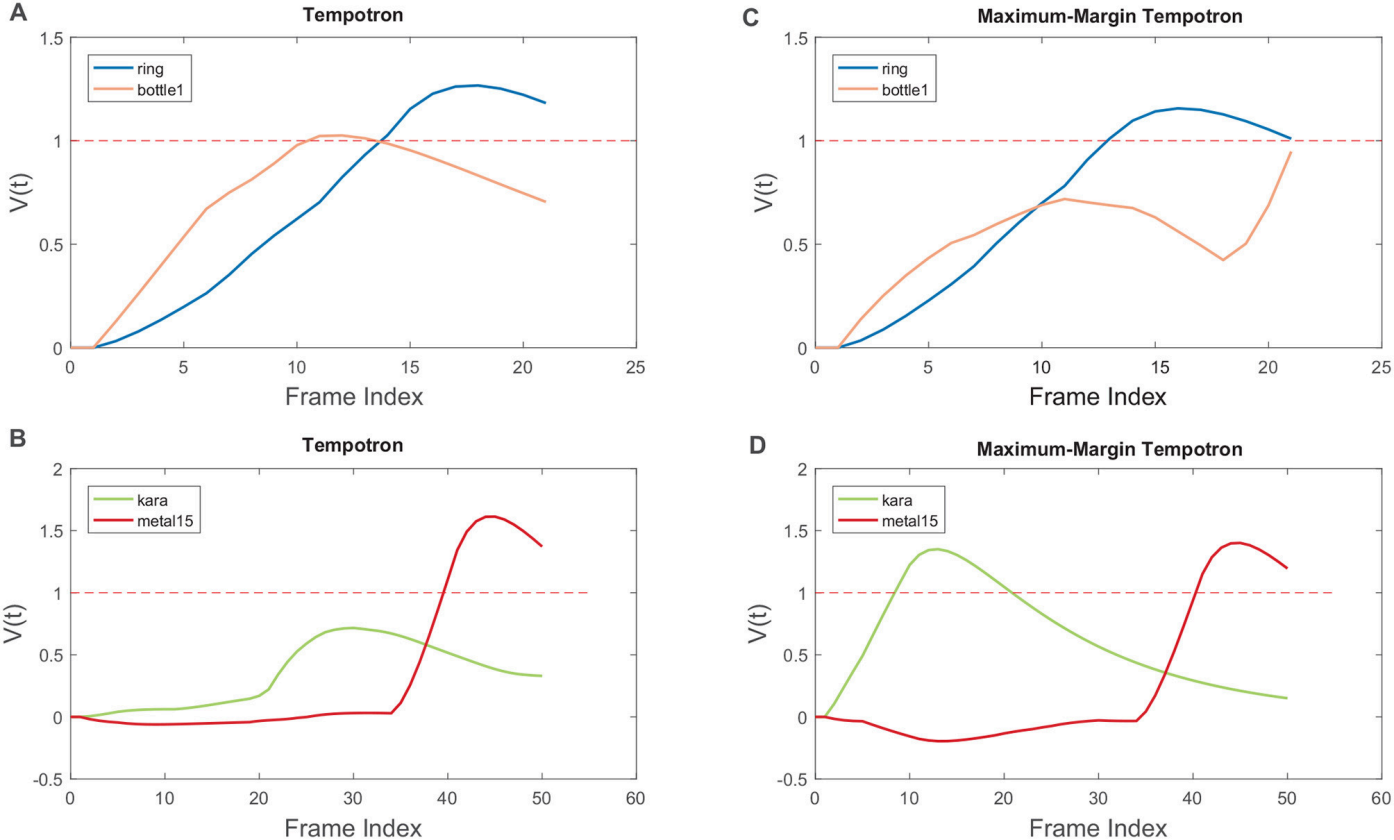
Selected samples misclassified by the Tempotron learning rule, while classified correctly by the modified Maximum-Margin Tempotron learning rule. Sample from the “ring” class misclassified as “bottle1” **(A)**, while correctly classified with Maximum-Margin Tempotron learning rule **(C)**. Sample from the “kara” class misclassified as “metal15” **(B)**, while correctly classified with Maximum-Margin Tempotron learning rule **(D)**.

To demonstrate how the hard margin Δ improves classification, we show two samples which have been misclassified by the SNN classifier trained with the original Tempotron rule (Figures 7A,B), but correctly classified by the Maximum-Margin Tempotron rule (Figures 7C,D). In Figure 7A, both output neurons (i.e., “ring” and “bottle1”) are selective to the discriminative local feature occurring between 2 and 10 ms. While in Figure 7B, the discriminative local feature is overlooked by the desired output neuron, possibly due to the time-warping, and the output neuron representing another class fires erroneously afterward.

When trained with the additional hard margin Δ, the negative output neuron representing the “bottle1” class is suppressed and prevented from firing (Figure 7C). Similarly, the negative output neuron representing the “metal15” class is also slightly suppressed, while the positive output neuron representing the “kara” class undergoes LTP and correctly crosses the *V*_*thr*_ (Figure 7D). Therefore, the additional hard margin Δ ensures a better separation between the positive and negative classes and improves classification accuracy.

**Figure 8:**
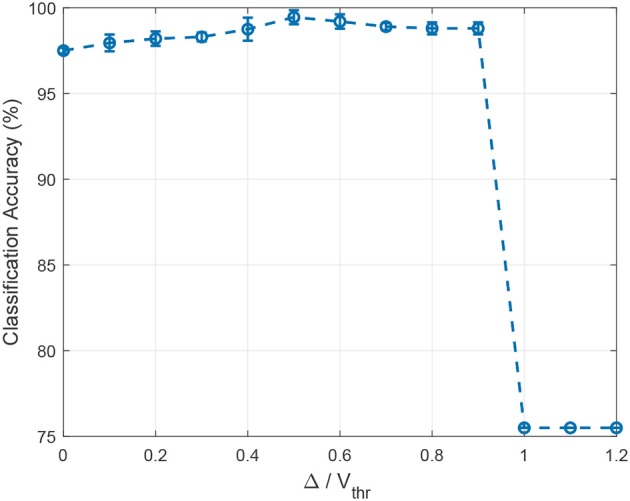
The effect of the ratio between the hard margin Δ and the firing threshold *V*_*thr*_ on classification accuracy. For Δ/*V*_*thr*_ = 0, the learning rule is reduced to the standard Tempotron rule. The hard margin Δ improves the classification accuracy for ratios below 1.0, while the accuracy drops significantly afterward. The best accuracy is achieved with a ratio of 0.5 on the RWCP dataset.

Since the relative ratio between the hard margin Δ and the firing threshold *V*_*thr*_ is an important hyper-parameter, we investigate its effect on the classification accuracy using the RWCP dataset by sweeping it from 0 to 1.2 with an interval of 0.1. The experiments are repeated 20 times for each ratio value with random weight initialization. For simplicity, we only study the symmetric cases whereby the hard margin has the same absolute value for both positive and negative neurons. For the case when the ratio is 0, the learning rule is reduced to the standard Tempotron rule. As shown in Figure 8, the hard margin Δ improves the classification accuracy consistently for ratios below 1.0, and the best accuracy is achieved with a ratio of 0.5. The accuracy drops significantly for ratio above 0.9, suggesting a high level of margin may interfere with learning and lead to brittle models.

### 3.5. Robustness to noise

#### 3.5.1. Environmental noise

We report the classification accuracies over 10 runs with random weight initialization in Tables 3, 4 for mismatched and multi-condition training respectively.

**Table 3:** Average classification accuracy of different models under the mismatched-condition.

**SNR**	**MLP**	**CNN**	**RNN**	**LSTM**	**SOM-SNN**
Clean	99.45 ± 0.35%	**99.85** **±** **0.23%**	95.35 ± 1.06%	98.40 ± 0.86%	99.60 ± 0.15%
20 dB	55.05 ± 4.30%	61.5 ± 4.71%	25.15 ± 8.86%	47.20 ± 5.36%	**79.15 ± 3.70%**
10 dB	32.10 ± 8.38%	**42.70** **±** **5.84%**	11.85 ± 2.06%	34.50 ± 10.61%	36.25 ± 1.25%
0 dB	24.60 ± 4.94%	**28.40** **±** **6.60%**	10.10 ± 1.64%	22.35 ± 6.63%	26.50 ± 1.29%
-5 dB	18.40 ± 4.58%	**22.65** **±** **5.08%**	9.20 ± 1.98%	16.60 ± 7.00%	19.55 ± 0.16%
Average	45.92%	51.02%	30.33%	43.81%	**52.21%**

*Experiments are conducted over 10 runs with random weight initialization*.

*The bold values indicate the best classification accuracies under different SNR*.

**Table 4:** Average classification accuracy of different models with multi-condition training.

**SNR**	**MLP**	**CNN**	**RNN**	**LSTM**	**SOM-SNN**
Clean	96.10 ± 1.18%	97.60 ± 0.89%	94.30 ± 3.04%	98.15 ± 0.71%	**99.80** **±** **0.22%**
20 dB	98.45 ± 0.61%	99.50 ± 0.22%	94.30 ± 2.70%	99.10 ± 0.89%	**100.00** **±** **0.00%**
10 dB	99.35 ± 0.45%	99.70 ± 0.33%	95.25 ± 2.49%	99.05 ± 1.25%	**100.00** **±** **0.00%**
0 dB	98.20 ± 1.45%	99.45 ± 0.75%	93.65 ± 2.82%	95.80 ± 3.93%	**99.45** **±** **0.55%**
-5 dB	92.50 ± 1.53%	98.35 ± 0.78%	86.85 ± 5.20%	91.35 ± 4.82%	**98.70** **±** **0.48%**
Average	96.92%	98.92%	92.87%	96.69%	**99.59%**

*Experiments are conducted over 10 runs with random weight initialization*.

*The bold values indicate the best classification accuracies under different SNR*.

We note that under the mismatched condition, the classification accuracy for all models degrades dramatically with an increasing amount of noise and falls below 50% with SNR at 10 dB. The LSF-SNN and LTF-SNN models use local key points on the spectrogram as features to represent the sound sample, and are therefore robust to noise under such conditions. However, the biological evidence for such spectrogram features is currently lacking.

As shown in Table 4, multi-condition training effectively addresses the problem of performance degradation under noisy conditions, whereby MLP, CNN, LSTM, and SOM-SNN models have achieved classification accuracies above 95% even at the challenging 0 dB SNR. Similar to observations made in McLoughlin et al. (2015), we note that the improved robustness to noise comes with a trade-off in terms of accuracy for clean sounds, as demonstrated in the results for the ANN models. However, the classification accuracies improve across the board for the SOM-SNN model under all acoustic conditions using the multi-condition training, achieving an accuracy of 98.7% even for the challenging case of -5 dB SNR. The SOM-SNN model hence offers an attractive alternative to other models especially when a single trained model has to operate under varying noise levels.

#### 3.5.2. Spike jittering

**Figure 9:**
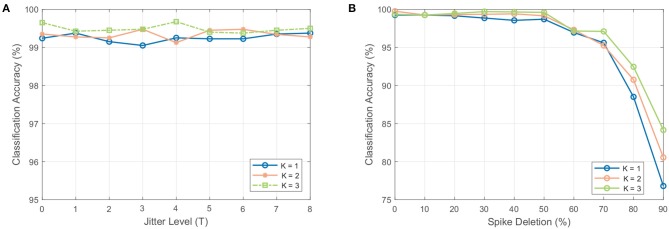
The effect of spike jittering and spike deletion on the classification accuracy. **(A)** Classification accuracy as a result of spike jitter added at the input to the SNN classifier. The amount of jitter is added as a fraction of the spike generation period *T* (i.e., 50 ms used for the RWCP dataset). The classifier is robust to spike jitter, maintaining a high accuracy with different amount of jitter. **(B)** Classification accuracy as a result of spike deletion at the input to the SNN classifier. The accuracy of the classifier remains stable for spike deletion ratio below 60% and decays with increased spike deletion.

As shown in Figure 9A, the SOM-SNN model is shown to be highly robust to spike jittering and maintains a high accuracy independent of the number of neurons activated per sound frame in the SOM. We suspect that given only a small subset of neurons in the SOM are involved for each sound class, the requirement of the SNN for precise spike timing is relaxed.

#### 3.5.3. Spike deletion

As shown in Figure 9B, the SOM-SNN model maintains a high classification accuracy when spike deletion is performed on the input to the SNN. As only a small subset of pre-synaptic neurons in the SOM deliver input spikes to the SNN for each sound class, with high inter-class variability, the SNN classifier is still able to classify correctly even with some input spike deletion. The peak membrane potential value is used in some cases to make the correct classification.

## 4. Discussion

In this paper, we propose a biologically plausible SOM-SNN framework for automatic sound classification. This framework integrates the auditory front-end, feature representation learning and temporal classification in a unified framework. Biological plausibility is a key consideration in the design of our framework, which distinguishes it from many other machine learning frameworks.

The SOM-SNN framework is organized in a modular manner, whereby acoustic signals are pre-processed using a biologically plausible auditory front-end, the mel-scaled filter bank, for frequency content analysis. This framework emulates the functionality of the human cochlea and the non-linearity of human perception of sound (Bear et al., 2016). Although it is still not clear how information is represented and processed in the auditory cortex, it has been shown that certain neural populations in the cochlear nuclei and primary auditory cortex are organized in a tonotopic fashion (Pantev et al., 1995; Bilecen et al., 1998). Motivated by this, the biologically plausible SOM is used for the feature extraction and representation of mel-scaled filter bank outputs. The selectivity of neurons in the SOM emerges from unsupervised training and organizes in a tonotopic fashion, whereby adjacent neurons share similar weight vectors. The SOM effectively improves pattern separation, whereby each sound frame originally represented by a 20-dimensional vector (mel-scaled filter bank output coefficients) is translated into a single output spike. The resulting BMU activation sequences are shown to have the property of low intra-class variability and high inter-class variability. Consequently, the SOM provides an effective and sparse representation of acoustic signals as observed in the auditory cortex (Hromádka et al., 2008). Additionally, the feature representation of the SOM was shown to be useful inputs for RNN and LSTM classifiers in our experiments.

Although the SOM is biologically inspired by cortical maps in the human brain, it lacks certain characteristics of the biological neuron, such as spiking output and access to only local information. Other studies (Rumbell et al., 2014; Hazan et al., 2018) have shed light on the feasibility of using spiking neurons and spike-timing dependent plasticity (STDP) learning rule (Song et al., 2000) to model the SOM. We would investigate how we may integrate the spiking-SOM and the SNN classifier for classification tasks in the future.

Acoustic signals exhibit large variations not only in their frequency contents but also in temporal structures. State-of-the-art machine learning based ASC systems model the temporal transition explicitly, using the HMM, RNN or LSTM, while our work focuses on building a biologically plausible temporal classifier based on the SNN. For efficient training, we use supervised temporal learning rules, namely the membrane-potential based Maximum-Margin Tempotron and spike-timing based ReSuMe. The Maximum-Margin Tempotron (combining the Tempotron rule with the maximum-margin classifier) ensures a better separation between the positive and negative classes, improving classification accuracy in our experiments. As demonstrated in our experiments, the SOM-SNN framework achieves comparable classification results on both the RWCP and TIDIGITS datasets against other deep learning and SNN-based models.

We further discover that the SNN-based classifier has an early decision making capability: making a classification decision when only part of the input is presented. In our experiments, the SNN-based classifier achieves an accuracy of 95.1%, significantly higher than those of the RNN and LSTM (25.7% and 69.2% respectively) when only 50% of the input pattern is presented. This early decision making capability can be further exploited in noisy environments, as exemplified by the cocktail party problem (Haykin and Chen, 2005). The SNN-based classifier can potentially identify discriminative temporal features and classify accordingly from a time snippet of the acoustic signals that are less distorted, which is desirable for an environment with fluctuating noise.

Environmental noise poses a significant challenge to the robustness of any sound classification systems: the accuracy of many such systems degrade rapidly with an increasing amount of noise as shown in our experiments. Multi-condition training, whereby the model is trained with noise-corrupted sound samples, is shown to overcome this challenge effectively. In contrast to the DNN and SVM classifiers (McLoughlin et al., 2015), there is no trade-off in performance for clean sounds in the SOM-SNN framework with multi-condition training; probably because the classification decision is made based on local temporal patterns. Additionally, noise is also known to exist in the central nervous system (Schneidman, 2001; van Rossum et al., 2003) which can be simulated by spike jittering and deletion. Notably, the SOM-SNN framework is shown to be highly robust to such noises introduced to spike inputs arriving at the SNN classifier.

The SNN classifier makes a decision based on a single local discriminative feature which often only lasts for a fraction of the pattern duration, as a direct consequence of the Maximum-Margin Tempotron learning rule. We expect improved accuracy when more such local features within a single spike pattern are utilized for classification, which may be learned using the multi-spike Tempotron (Gütig, 2016; Yu et al., 2018). The accuracy of the SOM-SNN model trained with the ReSuMe learning rule may also be improved by using multiple spike times. However, defining these desired spike times is a challenge exacerbated by increasing intra-class variability. Although the existing single-layer SNN classifier has achieved promising results on both benchmark datasets, it is not clear how the proposed framework may scale for more challenging datasets. Recently, there is progress made in training multi-layer SNNs (Lee et al., 2016; Neftci et al., 2017; Wu et al., 2018b), which could significantly increase model capacity and classification accuracy. For future work, we would investigate how to incorporate these multi-spike and multi-layer SNN classifiers into our framework for more challenging large-vocabulary speech recognition tasks.

For real-life applications such as audio surveillance, we may add inhibitory connections between output neurons to reset all neurons once the decision has been made (i.e., a winner-takes-all mechanism). This allows output neurons to compete once again and spike upon receipt of a new local discriminative spike pattern. The firing history of all output neurons can then be analyzed so as to understand the audio scene.

The computational cost and memory bandwidth requirements of our framework would be the key concerns in a neuromorphic hardware implementation. As the proposed framework is organized in a pipelined manner, the computational cost could be analyzed independently for the auditory front-end, SOM and SNN classifier. For the auditory front-end, our implementation is similar to that of the MFCC. As evaluated in Anumula et al. (2018), the MFCC implementation is computationally more costly compared to the spike trains generated directly from the neuromorphic cochlea sensor. Our recent work (Pan et al., 2018) proposes a novel time-domain frequency filtering scheme which addresses the cost issue in MFCC implementation. We expect the SOM to be the main computational bottleneck of the proposed framework. For each sound frame, the calculation of the Euclidean distance of synaptic weights from the input vector is done for each SOM neuron. Additionally, the distances are required to be sorted so as to determine the best-matching units. However, this computational bottleneck can be addressed with the spiking-SOM implementation (Rumbell et al., 2014; Hazan et al., 2018), whereby the winner neuron spikes the earliest and inhibits all other neurons from firing (i.e., a winner-takes-all mechanism) and hence by construction, the BMU. The spiking-SOM also facilitates the implementation of the whole framework on a neuromorphic hardware. In tandem with the SNN classifier, a fully SNN-based framework when implemented would translate to significant power saving.

As for memory bandwidth requirements, the synaptic weight matrices connecting the auditory front-end with the SOM and the SOM with the SNN classifier are the two major components for memory storage and retrieval. For the synaptic connections between the auditory front-end and the SOM, the memory bandwidth increases quadratically with the product of the number of neurons in the SOM and the dimensionality of the filter banks. Since the number of output neurons is equal to the total number of classes and hence fixed, the memory bandwidth only increases linearly with the number of neurons in the SOM. Therefore, the number of neurons in the SOM should be carefully designed for a particular application considering the trade-off between classification accuracy and hardware efficiency.

## Author contributions

JW performed all the experiments. All authors contributed to the experiments design, results interpretation and writing.

### Conflict of interest statement

The authors declare that the research was conducted in the absence of any commercial or financial relationships that could be construed as a potential conflict of interest.
